# Berberine Inhibits Breast Cancer Stem Cell Development and Decreases Inflammation: Involvement of miRNAs and IL-6

**DOI:** 10.1016/j.cdnut.2024.104532

**Published:** 2024-12-15

**Authors:** Nour Ibrahim, Nawal Alsadi, Hamed Yasavoli-Sharahi, Roghayeh Shahbazi, Mary Joe Hebbo, Darshan Kambli, Florencia Balcells, Chantal Matar

**Affiliations:** 1 Nutritional Sciences Department, Faculty of Health Sciences, University of Ottawa, Ottawa, Ontario, Canada; 2 Cellular and Molecular in Medicine Department, Faculty of Medicine, University of Ottawa, Ottawa, Ontario, Canada

**Keywords:** berberine, breast cancer, cancer stem cells, microRNAs, interleukin-6, epigenetics

## Abstract

**Background:**

Breast cancer (BC) is a health concern worldwide and is often accompanied by depressive symptoms in patients. In BC, elevated interleukin-6 (IL-6) levels contribute to an inflammatory signature linked to cancer stem cell (CSC) stemness and depressive behaviors. Bioactive food components, such as berberine (BBR), have preventative effects against BC by targeting CSCs.

**Objectives:**

This study aimed to investigate the effects of BBR on breast CSC proliferation, on levels of specific micro (mi)RNAs and IL-6 in vitro and in vivo, and in alleviating depressive-like behaviors in mice with BC.

**Methods:**

Mammosphere formation assays were conducted by treating murine 4T1 and human MDA-MB-231 BC cell lines with BBR. qPCR analysis of miRNAs miR-let-7c and miR-34a-5p was performed on 4T1 CSCs exposed to BBR. BBR was administered orally to female BALB/c, followed by injection with mammary carcinoma cells to induce BC. Behavioral tests were conducted to assess depressive-like behaviors. Tumor tissues were collected for ex vivo mammosphere assays, miRNA expression analysis, and IL-6 detection by ELISA. Serum was also collected for IL-6 analysis.

**Results:**

BBR treatment inhibited mammosphere formation and proliferation of CSCs derived from 4T1 and MDA-MB-231 cell lines. Quantification of mammosphere formation showed a significant decrease in both cell lines at 75 μM BBR (4T1: *P* < 0.001; MDA-MB-231: *P* < 0.0001). BBR upregulated the expression of miRNAs miR-let-7c and miR-34a in both cell lines, with miR-34a showing a significant increase (*P* < 0.001) and let-7c showing a significant increase (*P* < 0.05) in expression. In vivo, oral administration of BBR reduced mammosphere formation in breast tumor tissues (*P* < 0.0001) and elevated expression of miR-145 and miR-34a, with both showing significant upregulation (*P* < 0.0001), indicating its potential tumor-suppressive effects. BBR treatment resulted in a significant decrease in serum IL-6 levels (*P* < 0.05), suggesting anti-inflammatory properties, while the IL-6 in tumor tissue did not show significant changes (*P* > 0.05). However, no significant differences were observed in depressive-like behaviors between control and treatment groups.

**Conclusions:**

BBR may have the potential to be used as an “Epi-Natural Compound” to prevent cancer by reducing inflammation and affecting epigenetics.

## Introduction

Breast cancer (BC) is one of the most common types of cancer, accounting for a large number of cancer-related fatalities per year [1]. BC is the second-leading cause of cancer-related deaths among women globally, after lung cancer [2]. Despite this, BC remains the most often diagnosed malignancy in women, with an expected 2.1 million new cases reported in 2018 [2]. The developmental origins of health and disease hypothesis highlight the involvement of the environment in determining health outcomes, with a focus on the possibility of personalized treatments to promote lifelong health and disease prevention [[3], [4], [5]]. The epigenome is dynamic, allowing for short-term adaptation to the environment [[6], [7], [8]]. Interestingly, epigenetic alterations such as DNA methylation, histone modifications, and RNA are required for programming stem cells to differentiate into cellular and tissue lineages [9].

Cancer stem cells (CSCs) are tissue-specific stem cells that differentiate and self-renew, producing cancer cells [10]. Mutations that activate oncogenes or inactivate tumor suppressor genes can lead to the formation of CSCs [10]. CSCs exist in small populations, but they are assumed to be responsible for the growth and maintenance of a tumor, and they may enhance the likelihood of relapse in some malignancies [10]. CSCs are important targets in integrative oncology because they are resistant to standard cancer treatments [10]. Moreover, breast CSCs have the unique ability to create mammospheres, which are large groups of cells that give rise to tumor cells [11]. Therefore, mammosphere formation is considered a hallmark of BC [12]. Breast CSCs are being increasingly accepted as a primary cause of tumor growth; thus, they are one of the emerging new focuses of scientific research [12].

MicroRNAs (miRNAs) are tiny RNA molecules that regulate gene expression, which is often altered in cancer [13]. They silence specific genes by binding to mRNA, thereby affecting protein production [14]. This involvement in gene regulation makes them crucial for cell functions and cancer development [14]. Additionally, miRNAs act as epigenetic modulators, altering gene function without changing the gene sequence [15].

miRNAs are often dysregulated in cancer, with some miRNAs functioning as tumor suppressors and others functioning as oncogenes [16]. Oncogenic miRNAs are often overexpressed in cancer and act to increase tumor growth [14]. Tumor suppressor miRNAs are often downregulated in cancer, and their roles in maintaining normal biological processes are hindered [14]. miR-145 is a tumor suppressor miRNA [17]. The expression of transforming growth factor β1 is directly or indirectly regulated by miR-145, resulting in the inhibition of proliferation and migration of BC cells. Furthermore, it has been demonstrated that miR-145 targets key reprogramming factors such *OCT4*, *SOX2*, and *KLF4* to play a significant role in controlling cell differentiation [18,19]. Similarly, miR-34a functions as a tumor suppressor in BC by targeting genes that promote cell proliferation and survival. It inhibits the expression of oncogenes such as *cMyc*, *Bcl2*, and *Cdk4*, which slow tumor growth and cause cell cycle arrest [20]. Moreover, miR-let-7c is a tumor suppressor miRNA and is also a candidate diagnostic biomarker of major depression [21]. Let-7c has been found to be associated with CSCs and may be a potential therapeutic target for CSCs [22].

IL-6 signaling is implicated in the maintenance and expansion of CSC populations in BC [23]. IL-6 has a significant role in regulating inflammation and immune responses [23]. IL-6 may be involved in regulating stress, metabolism, and inflammation, leading to BC development [24]. Similarly, IL-6 has been linked to the development and progression of BC, with the IL-6 signaling pathway activated in cancer cells during cancer cell growth, angiogenesis, invasion, metastasis, and chemoresistance [25]. Targeting the IL-6 pathway has shown promise in BC treatment, with drugs developed to inhibit IL-6 activity or its receptor [25]. Research has also explored the potential of IL-6 as a biomarker for BC diagnosis, prognosis, and response to therapy [25,26]. In addition, a meta-analysis showed that depressed groups had more IL-6 levels than nondepressed groups [27].

Depressive symptoms are among the reported comorbidities in BC patients and have a negative impact on patients’ quality of life, disease progress, and overall survival [28,29]. Behavioral tests for evaluating depressive-like behavior in animal models are essential for preclinical research aimed at comprehending the neurobiological mechanisms underlying depression and testing prospective treatment approaches [30,31]. Among these tests are the tail suspension test (TST) and the forced swim test (FST) [30,31]. The FST, also called the Porsolt FST, compares the amount of time the animal spends swimming with its time floating in a tall, water-filled cylinder [30,31]. The TST is thought to reflect reduced motivation, which is a hallmark of depression in humans [30,31]. When an animal experiences the temporary, unavoidable discomfort of hanging by their tail, they will become motionless. Passive swaying is included in the definition of immobility, which is the lack of intentional movement [30,31].

An increasing variety of complementary and integrative medicines are being used as adjunct therapy and prevention against cancer [32]. Specifically, many plant-derived substances have been extensively studied to provide prevention strategies or as an addition to therapy for BC patients [33,34]. Berberine (BBR), naturally found in European barberry and tree turmeric, is an isoquinoline alkaloid, a yellow-colored, bitter-tasting vitamin [35,36]. It has potential therapeutic benefits, such as anti-inflammatory, antioxidant, antimicrobial, and antitumor effects, and can be applied in the treatment of treatment of cancer, cardiovascular disease, neurological disorders, and metabolic disorders such as diabetes and obesity [35,36]. BBR is able to induce apoptosis and cell cycle arrest through mechanisms of increasing cytochrome c, caspase-9, and cleavage of poly (ADP-ribose) polymerase while reducing levels of *Bcl2* [37]. BBR can alter the gene expression of various proteins involved in apoptosis and the cell cycle, which includes miRNAs [38]. Because BBR was shown to modulate the AMP-activated protein kinase (AMPK) pathway in a metformin-like way, and because metformin is known to have inhibitory effects on CSCs, we elected to shed light on the effect of BBR on CSCs and prevention of cancer [[39], [40], [41]]. Despite many studies depicting the beneficial effects of BBR in diabetes or cancer settings, the lack of data about its effects on CSCs, epigenetics, and BC remains.

The goal of this study was to determine the role of BBR in the mechanisms of prevention of BC and mitigating depression. Our specific objectives were to investigate the effect of BBR on breast CSC proliferation and on specific miRNA and IL-6 levels in vitro and in vivo and the effect of BBR in alleviating depressive-like behaviors in mice with BC.

## Methods

### In vitro experiments

#### BC cell culture

Murine 4T1 and human MDA-MB-231 cell lines obtained from the American Type Cell Collection were analyzed for this project. Both cell lines were cultured in Dulbecco’s modified Eagle’s medium (DMEM) medium supplemented with 10% fetal bovine serum (Sigma-Aldrich) and 1% penicillin/streptomycin. Cells were maintained at 37°C with 5% carbon dioxide in the humidified incubator.

#### Preparation of BBR solution for in vitro experiments

BBR was purchased from Sigma-Aldrich (BBR chloride hydrate) and dissolved in dimethyl sulfoxide to create a 5 mM stock. The cytotoxicity of BBR was determined by a lactate dehydrogenase (LDH) assay to determine cell viability. Both cell lines were seeded at a density of 5000 cells per well in a 96-well plate with various concentrations of BBR from 25 μM to 125 μM. The absorbance measurements from the LDH assay were linked with the cytotoxicity levels caused by BBR treatment. Cell cytotoxicity curves were plotted as a function of BBR concentration, and suitable statistical analyses were used to calculate the half-maximal inhibitory concentration.

#### Mammosphere formation in BC cell lines

4T1 and MDA-MB-231 cell cultures were placed in DMEM F12 medium supplemented with 500 μL L-glutamine, 500 μL sodium pyruvate, 250 μL hydrocortisone, 50 μL penicillin/streptomycin, 50 μL basic fibroblast growth factor (BFGF), 25 μL insulin, and 25 μL epidermal growth factor (EGF) to create CSCs. Cells (5 × 10^3^) treated with 5 concentrations of BBR (25 μM to 125 μM) were added to low-attachment-surface 96-well plates, and the control was the same number of cells without the addition of BBR. The cells were monitored, analyzed, and counted at 48 h for mammosphere formation under light microscopy. This experiment was repeated 3 times independently.

#### Real-time quantitative reverse transcription PCR for in vitro experiments

Total RNA was isolated from the 4T1 and MDA-MB-231 cell lines using TRIzol reagent after being exposed to 75 μM BBR for 48 h. This was done using the miRNeasy kit (Qiagen). The total RNA was treated with DNase I and reverse transcribed into cDNA. The cDNA was produced by Moloney murine leukemia virus reverse transcriptase (Invitrogen). qPCR was carried out to measure expression of miR-let-7c and miR-34a using TaqMan primers (Applied Biosystems) and a FastStart Taq Polymerase (Roche) in a CFX96 machine (Bio-Rad). The expression of SNORD-65 was used as an endogenous control.

### In vivo experiments

#### BBR preparation for oral administration to the mice

BBR was dissolved in distilled water. The study involved oral administration of BBR to mice through their drinking water. It is estimated that at this age, each mouse drinks 2 mL of water daily. A daily dosage of 125 mg/kg [42] was calculated based on the average weight of the mice, resulting in each mouse receiving 2.25 mg BBR daily. The dose used for BBR in the mice model is physiologically relevant based on published studies [43]. The dose was also in accordance with prior published studies of BBR in cancer models [39,42,44]. Careful handling procedures to maintain freshness and quality were followed. The solution was then stirred with a magnetic stir bar and submerged in a water bath at 37°C to ensure the complete dissolution of BBR. During the feeding regimen, control groups received bottles containing 1% sucrose water, while treatment groups received bottles containing BBR in 1% sucrose water. Water consumption was monitored every day to ensure proper intake of the treatment by observing the decrease of the water content from the measuring bottle; the water was estimated to be decreased by 6 mL daily in each cage containing 3 mice. All solutions were refrigerated promptly after use to maintain stability and efficacy.

#### Study design for in vivo experiment

Six- to 7-week-old female BALB/c mice (Charles River Laboratories) divided into 2 groups of 12 mice each. The timeline of the study is detailed in the Supplemental Figure 1. The experiment follows the protocol #HSe-4177 approved by the animal facility at the University of Ottawa. Three mice from the same group were housed together in the same cage with a 12-h light-dark cycle, consuming standard feed pellets diet. After acclimation of 1 week, the control group was given a daily dose of 1% sucrose water, and the treatment group was given a daily dose of 125 mg BBR/kg dissolved in 1% sucrose water for the entire experimental period until euthanasia. After 2 weeks of dietary intervention, all animals received injections with the 4T1 mammary carcinoma cell line (1400 cells/0.2 mL per mouse) into the mammary fat pad of the BALB-c mice to induce BC for in vivo experiments. Behavioral tests were performed on the mice, a TST and a FST separately, to assess depressive-like behaviors. After 3 wk of 4T1 cell injection, mice were ethically killed with a ketamine/xylazine cocktail. After euthanasia at 12 weeks of age, the tumor was collected and used for several experiments. The tumor was digested, CSCs were suspended for ex vivo mammosphere analysis, proteins were extracted for an ELISA test to assess the IL-6 concentration, and miRNAs were analyzed by qPCR. Serum was also collected from each mouse for IL-6 analysis by ELISA.

#### Ex vivo mammospheres

During euthanasia, tumor dissection entailed separating tumors into 3 sections, with the largest part specified for tumor dissociation and subsequent cell culture. Media for cell culture (100 mL) was prepared using DMEM F12 knock-out medium (Invitrogen) for stem cells (1000 μL of 100 mM sodium pyruvate [Sigma], 1000 μL of 100 mMl-glutamine, 500 μL of 100 μg/mL hydrocortisone, 100 μL of streptomycin/penicillin mixture (100 μg/mL and 100 IU/mL, respectively), 100 μL of 20 μg/mL BFGF, 50 μL of 10 mg/mL human insulin, and 50 μL of 20 μg/mL EGF. After the digestion process, the dissociated 4T1 cells from each sample were collected and seeded at a density of 5000 cells per well in 96-well plates (low attachment). Mammosphere growth and aspect were recorded at several time points (24, 48, and 72 h), including counting and microscope imaging to determine the impact of BBR on mammosphere development.

#### RNA extraction and qPCR analysis from tumor tissues

The second part of the tumor was designated for miRNA and PCR analysis. The miRNeasy kit and protocol were also used in this assay, as previously described. qPCR was carried out to measure the expression of miR-145 and miR-34a. The expression of SNORD-65 was used as an endogenous control.

#### IL-6 detection by ELISA kit in breast tumor tissue and serum

For protein extraction, a solution of protein kinase inhibitor and radioimmunoprecipitation assay lysis buffer was prepared. Tumor tissue was transferred to the lysis buffer-filled tubes for tissue lysis and protein extraction. The extracted proteins were isolated for further analysis and IL-6 detection using ELISA kit. Serum and tissue levels of the IL-6 cytokine were measured using the Mouse IL-6 Uncoated ELISA kit from Invitrogen. A Corning Costar 9018 ELISA plate was coated with capture antibody overnight at 4°C, followed by washing and blocking the wells to minimize nonspecific binding. Standard preparation and serial dilutions were carried out to create a standard curve. Subsequently, samples were added to the appropriate wells, followed by incubation. The detection antibody was then introduced, and after another round of washing, avidin-horseradish peroxidase was added. The plate underwent a final wash before the addition of 1× 3,3',5,5'-tetramethylbenzidine solution and incubation, followed by a stop solution. Absorbance at 450 nm was measured, with optional wavelength subtraction at 570 nm for data analysis.

#### Depressive-like behaviors

##### The forced swim test

For 6 mins, the mouse was submerged in a plastic cylinder that was 22 cm in diameter and 37 cm tall. The water temperature was 23°C. A camera recorded the mouse’s movements, which were then examined by the tracking program Ethovision (Noldus). The duration of immobility was the main target being assessed, and the immobility durations of both control and treatment groups were compared. Immobility was defined as the “inactive” state in which the animal was floating without any purposeful movement. The time spent in this immobile state during the last 4 min of the 6-min test was used as the primary outcome measure for depressive-like behavior.

##### The tail suspension test

Individual mice were suspended 50 cm above the floor by taping their tails to a horizontal bar for 6 min. The mice actively tried to escape, followed by periods of immobility. The total duration of immobility was calculated as the time that the force of the mouse’s movements was below a threshold criterion; the magnitude of the mouse’s movements was recorded in arbitrary units, with a baseline threshold set by the Behavioral Core at the University of Ottawa. For our system, the threshold value for immobility was set to 3 arbitrary units. This threshold was used to calculate the total duration of immobility for each mouse, with the time spent in an “inactive” state serving as the primary measure of depressive-like behavior.

### Statistical analysis

Statistical analysis was conducted using GraphPad Prism 5.0 software (GraphPad Software Inc). For the quantification of 4T1 and MDA-MB-231 mammospheres at different BBR concentrations, 1-way analysis of variance (ANOVA) followed by a Tukey multiple comparisons test was employed to determine statistical significance. Two-way ANOVA was used for the quantification of ex vivo mammospheres. The analysis of IL-6 serum and tissue concentration, miRNAs, and behavioral tests involved *t* tests and 1-way ANOVA to compare control and treatment groups. A significance level of *P* ≤ 0.05 was chosen, with the data presented as mean ± SEM.

## Results

### In vitro

#### Cytotoxicity

BBR had a negligible influence on cell growth when the concentration was <25 μM. However, when the concentration was between 50 and 75 μM, BBR inhibited 4T1 cell growth in a time- and dose-dependent manner. A dose higher than 100 μM was cytotoxic to the cells. The cytotoxicity of BBR in vitro was assessed using the LDH test, as shown in Supplemental Figure 2. Therefore, a BBR concentration equal to 75 μM was used in the remainder of the in vitro experiments.

#### CSC growth and proliferation

**FIGURE 1:**
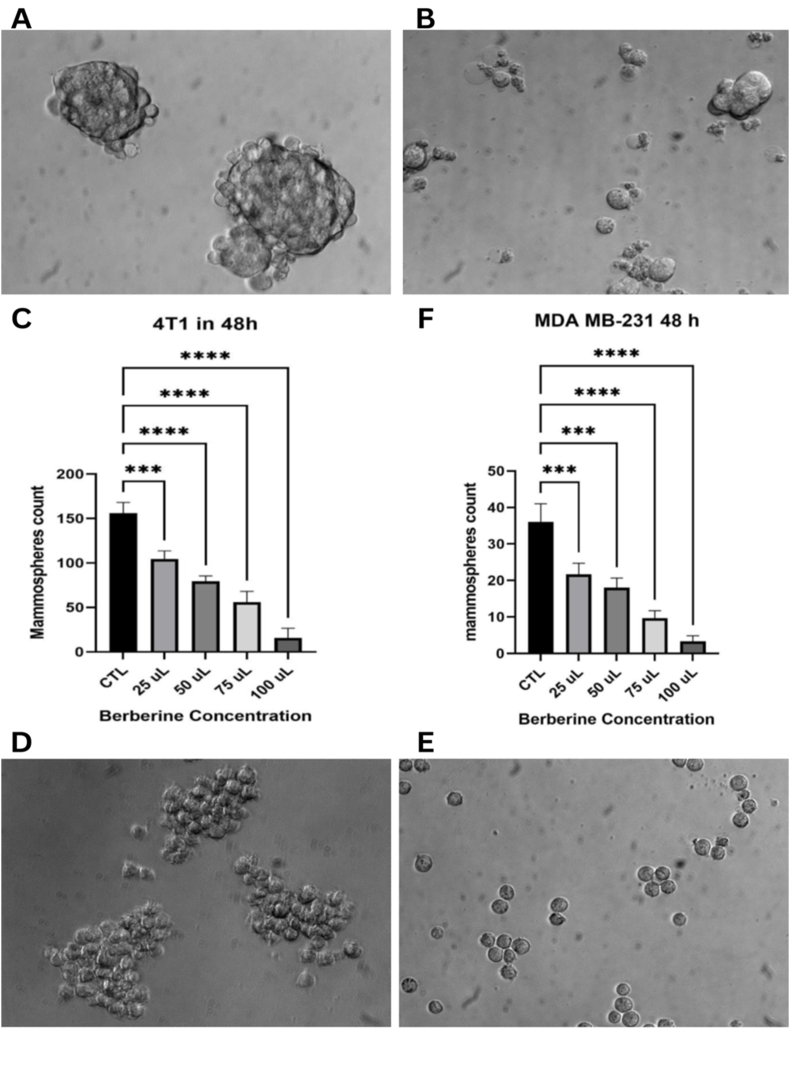
CSC growth and proliferation from both cell lines. (A) Untreated 4T1 CSCs (10× magnification). (B) 4T1 CSCs treated with 75 μM BBR (10× magnification). (C) Quantification of 4T1 mammospheres in different BBR concentrations. (D) Untreated MDA-MB-231 CSCs (10× magnification). (E) MDA-MB-231 CSCs treated with 75 μM BBR (10× magnification). (F) Quantification of MDA-MB-231 mammospheres in different BBR concentrations. Statistical significance was determined using a 1-way ANOVA and Tukey multiple comparisons test. ∗∗∗*P* < 0.001; ∗∗∗∗*P* < 0.0001. ANOVA, analysis of variance; BBR, berberine; CSC, cancer stem cell.

Figure 1 displays the results of 5 × 10^3^ CSCs extracted from both cell lines treated with 5 concentrations of BBR (0, 25, 50, 75, and 100 μM) counted and analyzed after 48 h. Mammospheres are formed significantly in the control group without BBR (Figure 1A). The treatment group (with 75 μM BBR) showed significantly smaller groups of cells as well as single cells but mammospheres were absent (Figure 1B). The same results were observed for the second cell line, MDA-MB-231 (Figure 1D, E). Mammosphere number was significantly decreased with the increase in BBR concentration in both cell lines (Figure 1C, F). This indicates the highly effective action of BBR in inhibiting mammosphere formation in breast CSCs. Thus, both BC cell lines were able to form mammospheres under non-adhering culture conditions, yet BBR treatment decreased mammosphere formation and proliferation.

#### Effect of BBR on miRNA expression in 4T1 cell culture

**FIGURE 2:**
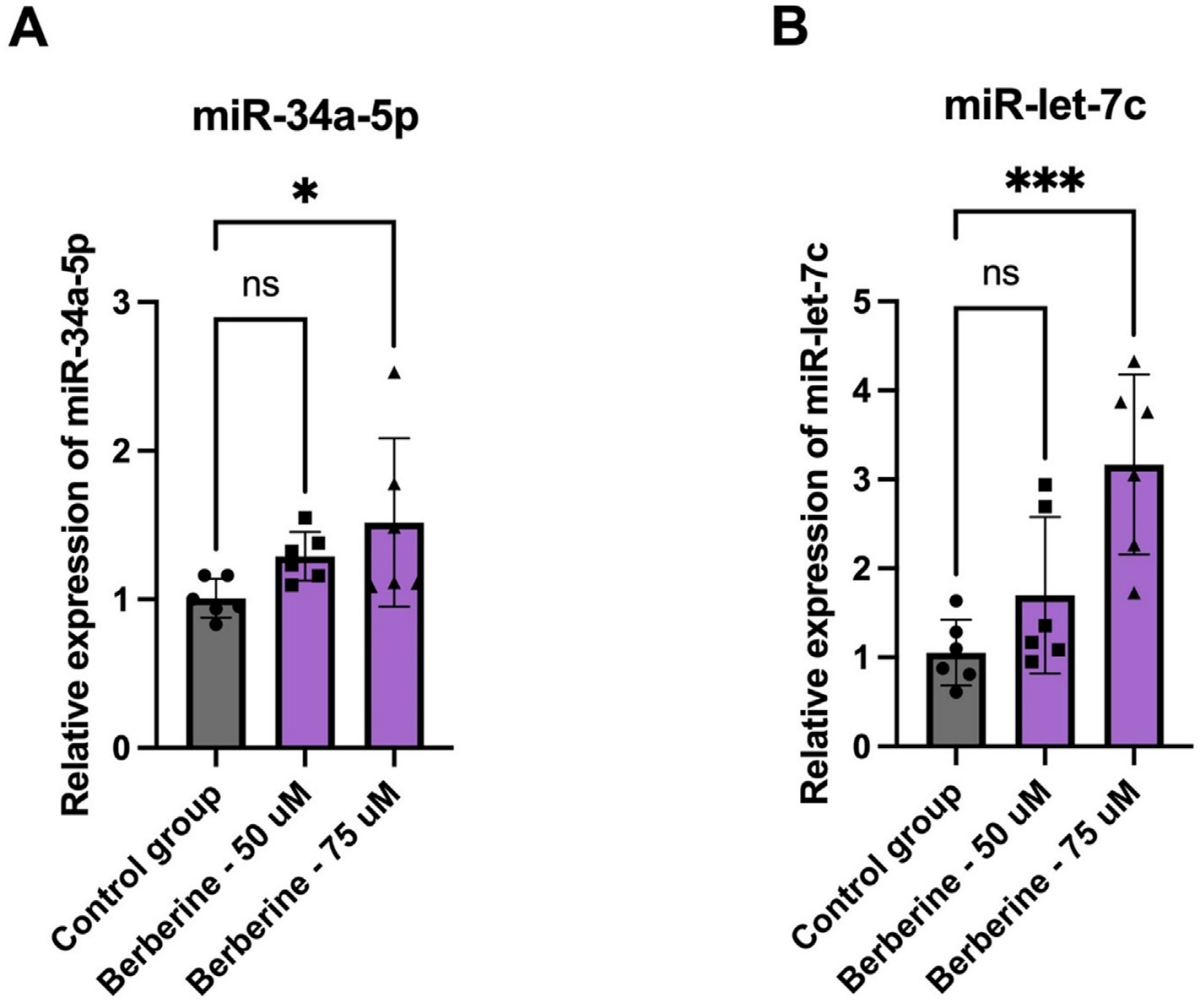
Relative expression of miRNAs in 4T1 cell culture in vitro. (A) miR-34a relative expression. (B) miR-let-7c relative expression. Results presented as mean ± SEM and statistically evaluated with a simple *t* test (∗*P* < 0.05; ∗∗∗*P* < 0.001).

miR-34a relative expression significantly increased in the 4T1 cells treated with BBR at a concentration of 75 μM compared to the untreated 4T1 cells control group, whereas it showed a nonsignificant elevation at a concentration of 50 μM compared to the control group (Figure 2A). Similarly, the expression of miR-let-7c significantly increased in the 4T1 cells treated with 75 μM BBR, whereas it showed a nonsignificant elevation at a concentration of 50 μM (Figure 2B). Thus, miR-34a and miR-let-7c are upregulated by BBR.

### In vivo

At week 12, 10 mice developed hard mammary tumors in the control group, and 12 mice developed tender mammary tumors in the treatment group. The mice were then killed. The average weights of the mice in the control and treatment groups were 19.79 g and 20.1 g respectively. The average tumor weight and dimensions for the control groups were 140.9 mg and 0.249 cm^3^, respectively, compared to the treatment group with 184.16 mg and 0.312 cm^3^ respectively.

#### Ex vivo mammospheres: CSC growth and proliferation

**FIGURE 3:**
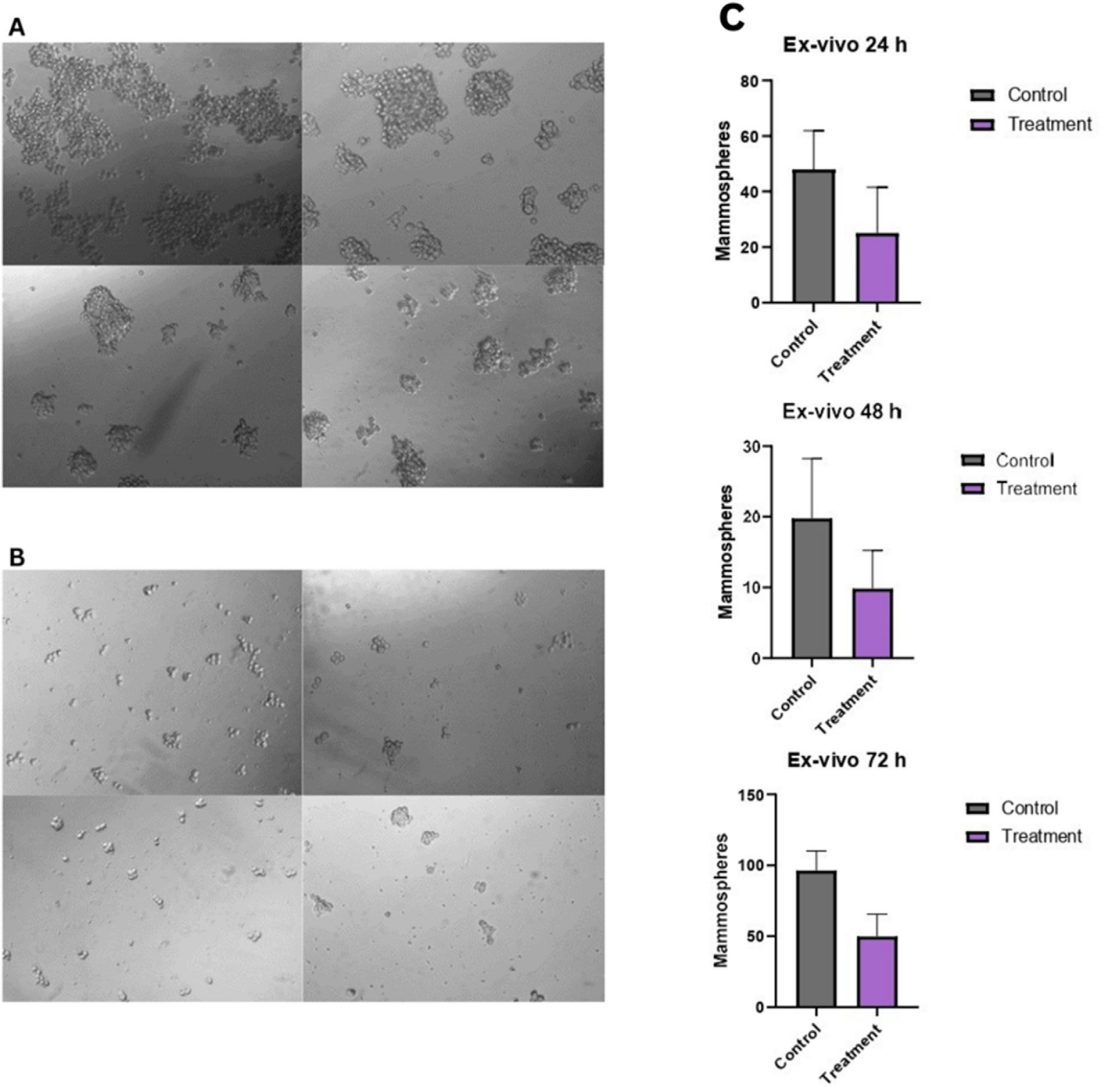
Ex vivo mammospheres of the breast tumors both groups. (A) Microscope image of mammospheres (10× magnification) from untreated mice consuming 1% sucrose water injected with the 4T1 cell line. (B) Mammospheres of BBR-treated mice consuming BBR solution injected with the 4T1 cell line. (C) Ex vivo mammosphere quantification of the breast tumors in the control and treatment groups; results presented as mean ± SEM. Statistical significance was determined using a simple *t* test.

For this experiment, CSCs were extracted from breast tumors without any further treatment. Figure 3A displays the results of 5 × 10^3^ concentrations of cells from the control group counted after 72 h. Figure 3B displays the results of 5 × 10^3^ concentrations of cells from the treatment group counted after 72 h. Comparing both mammosphere formations, the control mammospheres were more clustered and larger in number. In the treated group, mammospheres were in smaller clusters and more disintegrated, and single cells were also present. There was a significant decrease in ex vivo mammospheres in the treated group compared with the control group (Figure 3C). Thus, BBR inhibits mammosphere formation and proliferation in breast CSCs.

#### Effect of BBR on miRNA expression in the tumor

**FIGURE 4:**
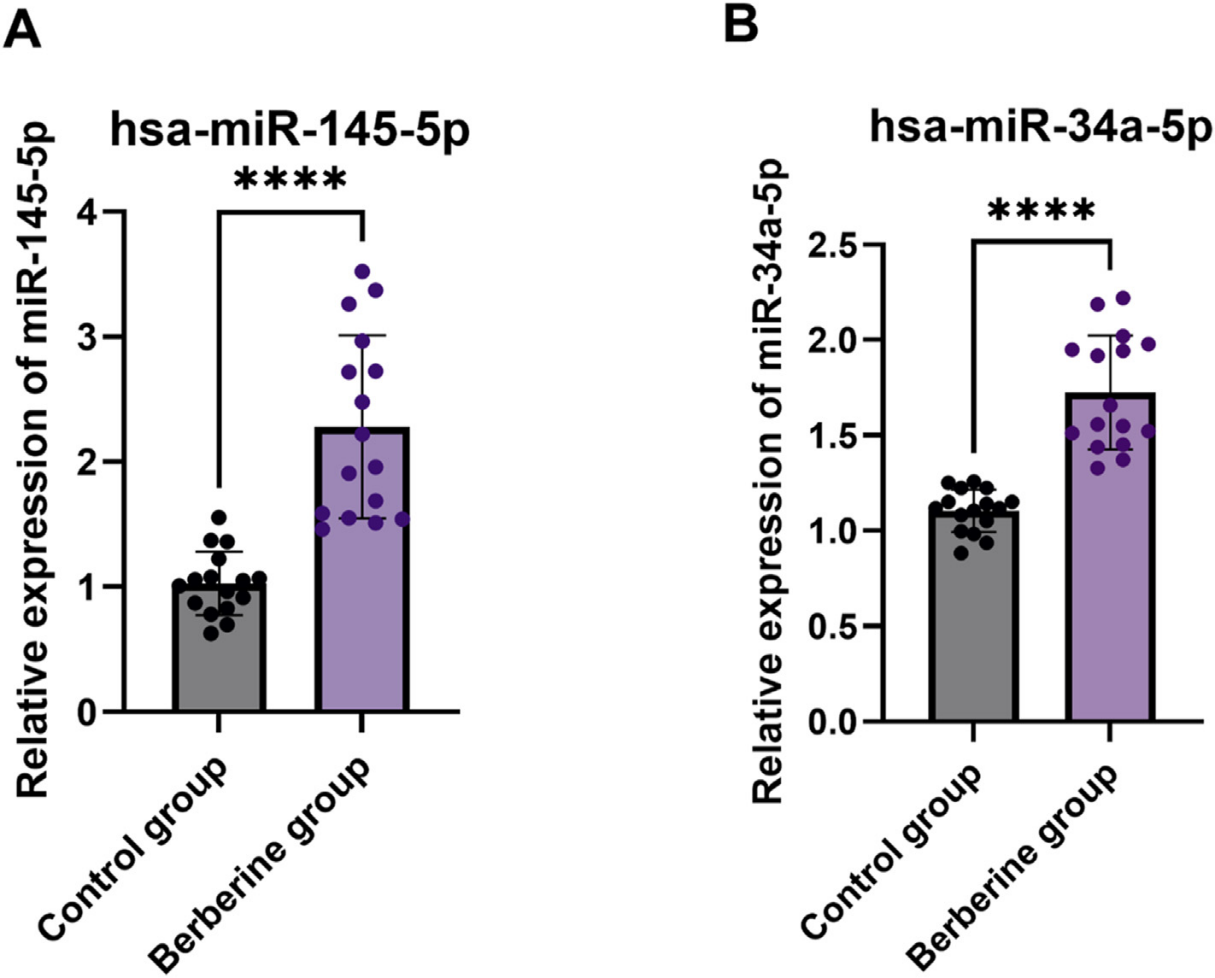
Relative expression of miRNAs in tumor tissues. (A) miR-145 relative expression in breast cancer tumor tissues. (B) miR-34a relative expression in breast cancer tumor tissues. Results presented as mean ± SEM and statistically evaluated with a simple *t* test (∗∗∗∗*P* < 0.0001).

The data showed a significant elevation in the expression of miR-145 in the BBR-treated group compared with the untreated control group (Figure 4A). Similarly, there was a significant elevation in the expression of miR-34a in the BBR-treated group compared with the untreated control group (Figure 4B). This suggests that BBR significantly upregulates the expression of miR-145 and miR-34a in BC tumors.

#### IL-6 detection in serum and in breast tumor tissue

**FIGURE 5:**
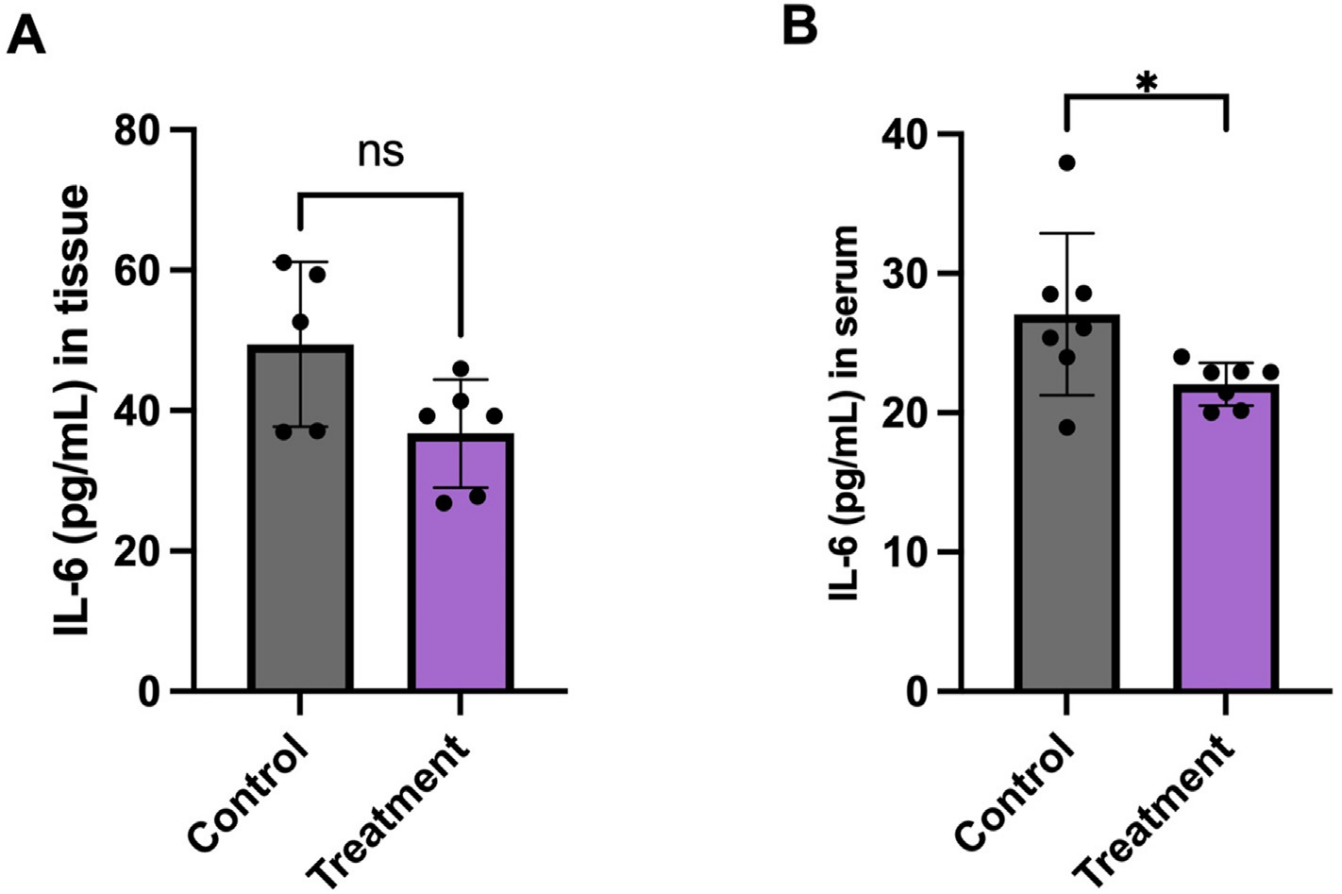
IL-6 levels measured with ELISA. **(A)** IL-6 levels in BALB-c mouse breast tumor. (B) IL-6 levels in BALB-c mouse plasma pools (pg/mL). The graphs show the mean ± SEM; statistical significance was determined using *t* test analysis (∗*P* < 0.05; ns, no significance).

Oral administration of BBR for 5 wk led to a trend but a nonsignificant reduction in the proinflammatory cytokine IL-6 levels in the breast tumor tissue of the BBR-treated group compared with the control group (*P* > 0.05) (Figure 5A). Similarly, there was a significant reduction in the proinflammatory cytokine IL-6 levels in the serum of the BBR-treated group compared with the control group (*P* < 0.05) (Figure 5B).

#### Depressive-like behaviors

**FIGURE 6:**
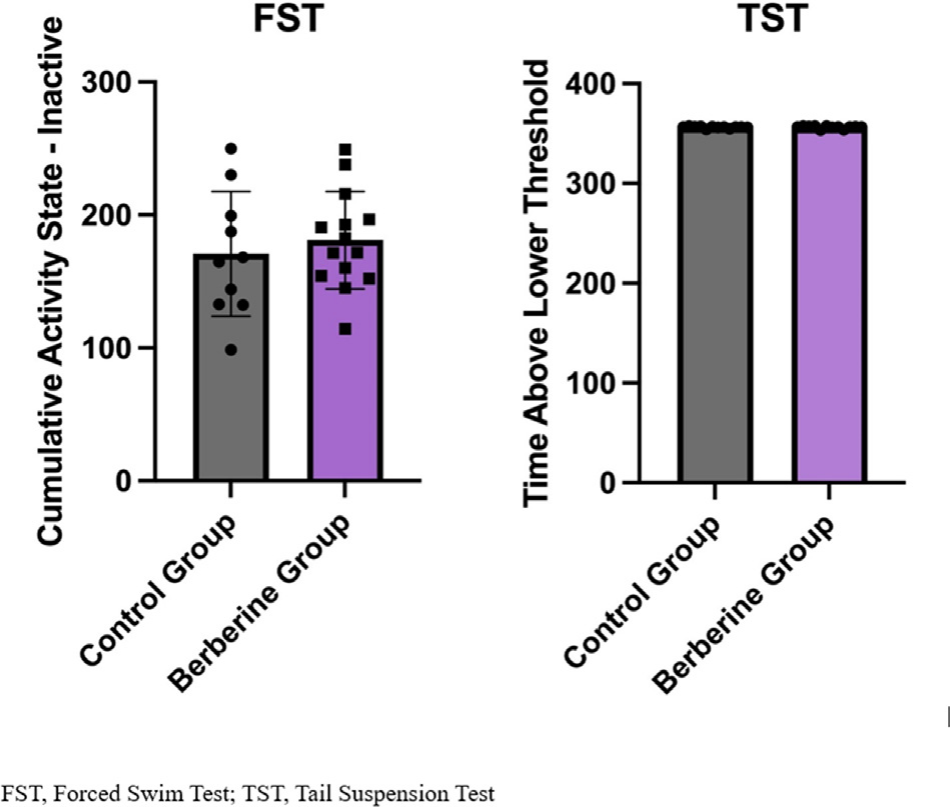
Depressive-like behavior tests in female mice exposed to either 1% sucrose water or berberine in 1% sucrose water treatment at week 11 of age. (A) The duration of inactivity in seconds (sec) during the forced swim test (FST). (B) The duration below the threshold in the tail suspension test (TST). Data are presented as mean (± SEM); no significant difference between groups; *n* = 12 for each group.

For the FST, using the Ethovision data, based on the length of immobility, which was measured as the cumulative duration of “activity state inactive,” the average periods of immobility for every experimental group were compared. The analysis did not show any statistically significant differences between the control and treatment groups (*P* > 0.05) (Figure 6A). Similarly, for the TST, the total amount of time the mouse spent below the activity threshold was the target value to assess depressive-like behaviors. The activity threshold is set by the Behavioral Core at the University of Ottawa based on it and is considered “inactivity” in this test. The data has no units; it is an arbitrary value calculated relative to the baseline. The analysis of the average “time below the lower threshold” revealed no statistically significant difference between the control and the BBR group (*P* > 0.05) (Figure 6B).

## Discussion

Breast carcinogenesis involves dysregulation of tumor suppressor genes and oncogenes. This study investigates the effects of BBR on BC and depressive-like behaviors in mice including miRNAs and IL-6. The results showed that BBR exhibits promising effects in BC therapy and prevention by targeting multiple pathways involved in tumor progression and inflammation modulation.

As seen in this study, BBR targeted CSCs by decreasing mammosphere formation and proliferation. CSCs are implicated in tumor initiation, recurrence, metastasis, and resistance to therapy [10,45]. The ability of BBR to reduce sphere formation in CSCs may be associated with its ability to induce apoptosis. In a previous study, BBR liposomes were found to induce apoptosis of CSCs by increasing levels of proapoptotic proteins such as Bax and decreasing those of antiapoptotic proteins such as Bcl-2 [46]. It was shown that BBR was able to target the mitochondria of breast CSCs to induce apoptosis [46]. Furthermore, the AMPK pathway may contribute to the ability of BBR to inhibit the growth of CSCs. Previous research has found CSCs to be sensitive to the AMPK pathway [22]. Moreover, BBR has been shown to have the ability to modulate the AMPK pathway [22,46]. The AMPK pathway is involved in the regulation of the cell cycle, cell proliferation, and cell survival [22]. A previous study found BBR was able to stimulate the AMPK pathway and, as a result, inhibit the mTORC1 pathway, which is involved in cell proliferation [22]. In addition, BBR was able to inhibit metastasis through regulation of the AMPK pathway and inhibition of certain matrix metalloproteinases (MMPs) in another study [46]. In regulating the AMPK pathway, BBR exhibits anti-inflammatory and antioxidant effects [46].

In this study, the targeted miRNAs were significantly increased in BBR-treated groups compared with the control group, both in vitro and in vivo. Because miR-145, miR-34a, and miR-let-7c are frequently dysregulated in BC, they act as tumor suppressors by targeting critical oncogenes that control cell proliferation, apoptosis, and metastasis [20,47]. Dysregulation of these miRNAs is linked to BC progression, aggressiveness, and resistance to treatment.

miR-145 targets cell reprogramming genes such as *Oct4* and *Sox2*, which are implicated in CSC maintenance [18]. It inhibits human embryonic stem cell regeneration, promotes differentiation, and plays a significant role in carcinogenesis and tumor development [18]. Elevated *Oct4* levels are regarded as one of the most important indicators of cancer cell chemotherapy resistance; however, increasing miR-145 levels can reduce expression of *Oct4* and its target gene *Zeb1*, counteracting the increase in *Oct4* levels generated by pemetrexed treatment, a type of chemotherapy [48]. Restoration of miR-145 expression can efficiently increase apoptosis in BC cells expressing wild-type *TP53* or estrogen receptor, implying that miR-145 therapy may be helpful in patients with BCs expressing wild-type *TP53* or estrogen receptor [49]. Moreover, miR-145 may inhibit cancer cell proliferation by targeting genes associated with growth factors, including *IRS-1*, *IGF-IR*, or epidermal growth factor receptor (*EGFR*) [50,51]. Meanwhile, miR-145 may inhibit the angiogenesis process by targeting N-RAS, VEGF, or hypoxia-inducible factor 1α (HIF-1α) [52,53]. A prior study found that BBR increased miR-145 expression while decreasing MMP16 expression, reducing the proliferation, migration, and metastasis of ovarian cancer SKOV3 and 3AO cells [12]. Another study on ovarian cancer showed that BBR inhibited the Warburg effect via the TET3/miR-145/HK2 pathways in ovarian cancer cells [54].

In this study, miR-let-7c was significantly increased in BBR-treated groups. Previously, miR-let-7c contributed significantly to cell apoptosis and cell growth suppression in BC, in part by targeting ERCC6 [55]. miR-let-7c acts as a cancer suppressor in a variety of ways, including preventing early cancer progression by suppressing *Hmga2* expression [56], inhibiting migration and invasion of human non-small cell lung cancer and colorectal cancer [57,58], and inducing cell apoptosis and disrupting the cell cycle in human hepatocellular carcinoma cells. In BC patients, miR-let-7c is downregulated in both tissues and serum, and postmenopausal status influences miR-let-7c expression [59]. A higher expression level of miR-let-7c has been linked to a better clinical outcome in individuals with estrogen receptor-positive BC [60].

In this study, miR-34a was significantly increased in BBR-treated groups both in vitro and in vivo. In a recent study, BBR was shown to have a unique antidepressant-like mechanism that inhibits miR-34a to restore synaptotagmin-1 and Bcl-2 expression, resulting in improved spinal morphology, mitochondria, and neurogenesis in the hippocampus [61]. This increase of expression is linked to miR-34a function as a tumor suppressor miRNA and is frequently downregulated in many malignancies [20,[62], [63], [64]]. It is recognized to have an important role in causing apoptosis, or programmed cell death. miR-34a promotes apoptosis by targeting genes involved in cell survival, proliferation, and antiapoptotic pathways [65]. Several studies have shown that miR-34a overexpression can induce apoptosis in cancer cells via many pathways [64]. For example, miR-34a targets genes such as *Bcl2*, an anti-apoptotic protein [66]. miR-34 inhibits *Sirt1* deacetylase implicated in cell survival pathways; it increases acetylated p53 and expresses p21 and PUMA, p53 transcriptional targets that regulate the cell cycle and apoptosis, respectively [67]. miR-34a-5p may play a significant role in initiating apoptosis by downregulating *Snai1* in apigenin-treated lung cancer cells [20]. miR-34a causes apoptosis while inhibiting tumor growth by downregulating these genes. Additionally, miR-34a has been linked to the regulation of p53, a master regulator of apoptosis and tumor suppression. miR-34a is a p53 transcriptional target, resulting in a feedback loop that amplifies apoptotic induction in response to cellular stress [64]. miR-34a is located on 1p36 and is typically deleted in neuroblastomas. Furthermore, a reduction in miR-34 expression has been associated with resistance to apoptosis caused by p53-activating substances used in chemotherapy [62,64]. Another study emphasized how miR-34a-5p and HOX transcript antisense RNA (HOTAIR) interact to control genes linked to the epithelial–mesenchymal transition in lung cancer cells. As a competing endogenous RNA, HOTAIR binds to miR-34a-5p and stops it from regulating snail, which subsequently suppresses E-cadherin. Targeting miR-34a-5p and HOTAIR may be a viable therapeutic approach to regulate non-small cell lung cancer development and metastasis, as the combination of BBR and gefitinib intensifies the inhibition of this pathway [68]. Its capacity to induce apoptosis emphasizes its utility as a diagnostic and therapeutic agent in cancer treatment.

IL-6 levels in the serum are reduced by BBR. IL-6 signaling is implicated in the maintenance and expansion of CSC populations in BC [25]. IL-6 is also essential for epigenetic alteration in stem cells [69,70]. IL-6 activates the NF-κB and STAT3 signaling pathways [71,72]. NF-κB and STAT3 have been recognized as major regulators of epigenetic switches in inflammation [73,74]. Recently, a positive feedback loop involving miRNA let-7 has been shown to maintain chronic inflammation in malignant cells [74]. The feedback loop mediated by IL-6 signaling can activate the NF-κB pathway and its downstream targets, including let-7 and Lin-28. Similarly, IL-6 was shown to be critical in maintaining the inflammatory loop in breast CSCs [73,74]. In summary, IL-6 signaling regulates cancer cell proliferation, CSC renewal, and metastasis. CSCs, in turn, produce IL-6, creating a positive feedback loop that sustains CSC populations within the tumor microenvironment [75]. Although it also affects a variety of nonimmune cells, such as fibroblasts and endothelial cells that express gp130 during inflammation, IL-6 primarily works on lymphoid and myeloid immune cells that express both IL-6Rα and gp130 [76]. Moreover, IL-6 and IL-17 or TNFα synergistically promote the production of numerous proinflammatory mediators including IL-6 from nonimmune cells, which is dependent on STAT3 and NF-κB [77]. Inflammatory disorders, autoimmune diseases, and cancer are caused by intricate interactions between nonimmune and immune cells via the IL-6 Amp. As a result, the expression and/or activities of IL-6 Amp-related molecules may be used to anticipate diseases caused by local initiators [76]. Targeting IL-6 signaling pathways may disrupt CSC function and sensitize breast tumors to conventional therapies, potentially improving patient outcomes.

The absence of significant results in the FST and TST between the control and treatment groups could be attributed to the study’s limited duration or the aggressiveness of the breast tumor, which hid the effect of BBR on depressive-like behaviors in mice by not allowing enough time for behavioral changes. This means the third hypothesis was not confirmed, and further research is required.

There were no significant results found regarding depressive-like behaviors. The lack of significant findings in both the TST and FST depressive-like behaviors could be attributed to several factors. One potential explanation is the relatively short time frame for behavioral assessment following the treatment. In this study, we used the aggressive 4T1 BC model, which requires a 3-wk period before euthanasia due to the rapid tumor progression. This timeline may have been insufficient to capture the full spectrum of depressive-like behaviors, as chronic stress and tumor progression can take time to manifest in behavioral alterations. It is possible that a longer duration of treatment and observation would have provided a clearer picture of the behavioral effects. Another factor to consider is the specific nature of the cancer model used. The 4T1 model is known for its high aggressiveness, which may result in significant physiological stress, but this may not necessarily translate into measurable depressive-like behaviors as assessed by the TST and FST. However, our study showed the potential of BBR on decreasing IL-6 levels in serum, and it was previously stated that IL-6 plays a role in stress-related psychiatric disorders, proposing that increased gut permeability may lead to the translocation of gut bacteria and their metabolites into the bloodstream, which can activate an immune response and cause inflammation (neuroinflammation) and the implication of the proinflammatory cytokine IL-6 that impacts the brain, resulting in mood and behavioral changes [78,79]. Thus, we believe further investigations are needed regarding the third hypothesis.

The limitations of this study include the mammosphere formation assays. Although these assays provide valuable insights into cancer stemness, they are primarily an initial approach. The study would benefit from incorporating complementary assays that examine additional markers, such as CD49f, to provide a more comprehensive understanding of CSC characteristics and behavior [80]. The 4T1 cancer model used in this study presents several limitations. First, it is an aggressive, highly metastatic model, which may not fully reflect the progression of all types of human BC, particularly less aggressive forms. The rapid tumor growth in this model limits the ability to study early tumor development and the longer-term effects of treatments. Additionally, the 3-wk period before euthanasia may not have allowed enough time for observing the full range of behavioral changes, particularly in terms of depressive-like behaviors. This relatively short duration might not capture the delayed onset of some cancer-related symptoms, including depression, which could require more extended periods of observation. Furthermore, although the 4T1 model is valuable for testing therapeutic interventions, it does not account for the heterogeneity observed in human BC. Although IL-6 levels were reduced in the treated group, additional confirmation by IL-6 and other proinflammatory cytokines in the brain would provide a more complete knowledge of neuroinflammatory alterations, increasing the depth of the study. The inclusion of a negative control group without BC would have improved the study’s comparability, especially in behavioral measures of depressive-like behaviors. Although 1% sucrose may appear nonsignificant, the cumulative effect over a 5-wk period could potentially affect the microbiota composition and other physiological factors in mice. However, the sugar addition to both groups was intended to hide the bitter taste of BBR for more palatability and to ensure ingestion.

Although our study has some limitations, it also has considerable strengths. First, our comprehensive experimental design includes both in vitro and in vivo tests, allowing for a thorough investigation of the effects of BBR on breast CSC and depressive-like behaviors. This multimodal approach not only strengthens our findings but also provides a more complete picture of the underlying mechanisms. Furthermore, our findings are significant, providing valuable insights into the potential therapeutic or preventative use of BBR in BC. Moreover, to ensure objectivity and minimize bias, other colleagues were blinded to both groups while analyzing our results for confirmation, further enhancing the credibility of our findings.

The current research suggests various prospects for advancement in the future. Extending the period of behavioral assessments, particularly with a less aggressive cancer cell line, may provide a more complete knowledge of the effect of BBR on depressive-like behaviors throughout time. A less aggressive model would allow more time to evaluate the progression of depressive symptoms and the potential impact of BBR on mood regulation over a longer period. Additionally, a longer experimental timeline could give more insight into the cumulative effects of cancer and treatment on depressive-like behavior. It is also possible that chronic stress associated with slower tumor progression in a less aggressive model may more closely mimic the gradual onset of depressive symptoms observed in cancer patients, providing a more nuanced understanding of the impact of BBR on this aspect of cancer-related comorbidities. Evaluating short-chain fatty acids in the feces of BBR-treated mice could help to understand its prebiotic properties and their implications for gut microbiota modification. Furthermore, undertaking microbiome profile research, for example, by fecal analysis, provides a chance to identify specific microbial signatures altered by BBR, clarifying its therapeutic potential. Decreasing apoptosis through cell cycle and apoptosis assays to verify the mechanism that BBR follows to decrease CSCs is also a good approach to consider in future research. Pursuing these prospective initiatives offers potential for expanding our understanding of the effects of BBR on BC, depressive behaviors, neuroinflammation, and gut microbiota, ultimately leading to better preventative measures, therapeutic techniques, and patient outcomes. These mechanisms suggest that BBR may enhance the effectiveness of conventional cancer therapies, such as chemotherapy and targeted therapies, However, clinical trials are needed to validate these findings, determine the most effective dosing regimens, and assess the safety and efficacy of BBR in combination with existing cancer treatments in cancer patients.

In conclusion, the results of this study suggest the potential role of BBR in preventing BC stem cell formation and proliferation by modulating IL-6 and miRNA expression. Therefore, these substances may have the potential to be used as “Epi-Natural Compounds” to prevent BC. It is crucial that further studies are conducted to confirm these results and search further into the role of BBR as an adjunct to traditional BC therapy. These findings may verify the antitumor characteristics of BBR as a novel next-generation prebiotic.

## Author contributions

The authors‘ responsibilities were as follows – NI: performed experiments, wrote the manuscript, prepared the figures, and performed data analysis; NA, HY-S, RS: taught all the techniques and helped in statistical analysis; MJH, DK, FB: collected samples; CM: designed and supervised the work; and all authors: read and approved the final manuscript.

## Funding

This research received Nutrition and Mental Health Scholarship and a Merit Scholarship from the University of Ottawa. This research was funded - Exploration NFRFE-(2019-01497) and Cancer Research Society CRS-(943594).

## Conflict of interest

The authors report no conflict of interest.
