# Supplementary material for: Berberine Inhibits Breast Cancer Stem Cell Development and Decreases Inflammation: Involvement of miRNAs and IL-6
Source: Curr Dev Nutr. 2024 Dec 15;9(2):104532. doi: 10.1016/j.cdnut.2024.104532 (PMC11786844; doi:10.1016/j.cdnut.2024.104532)
Supplement: Multimedia component 1 [file mmc1.docx]

Supplementary Figure 1


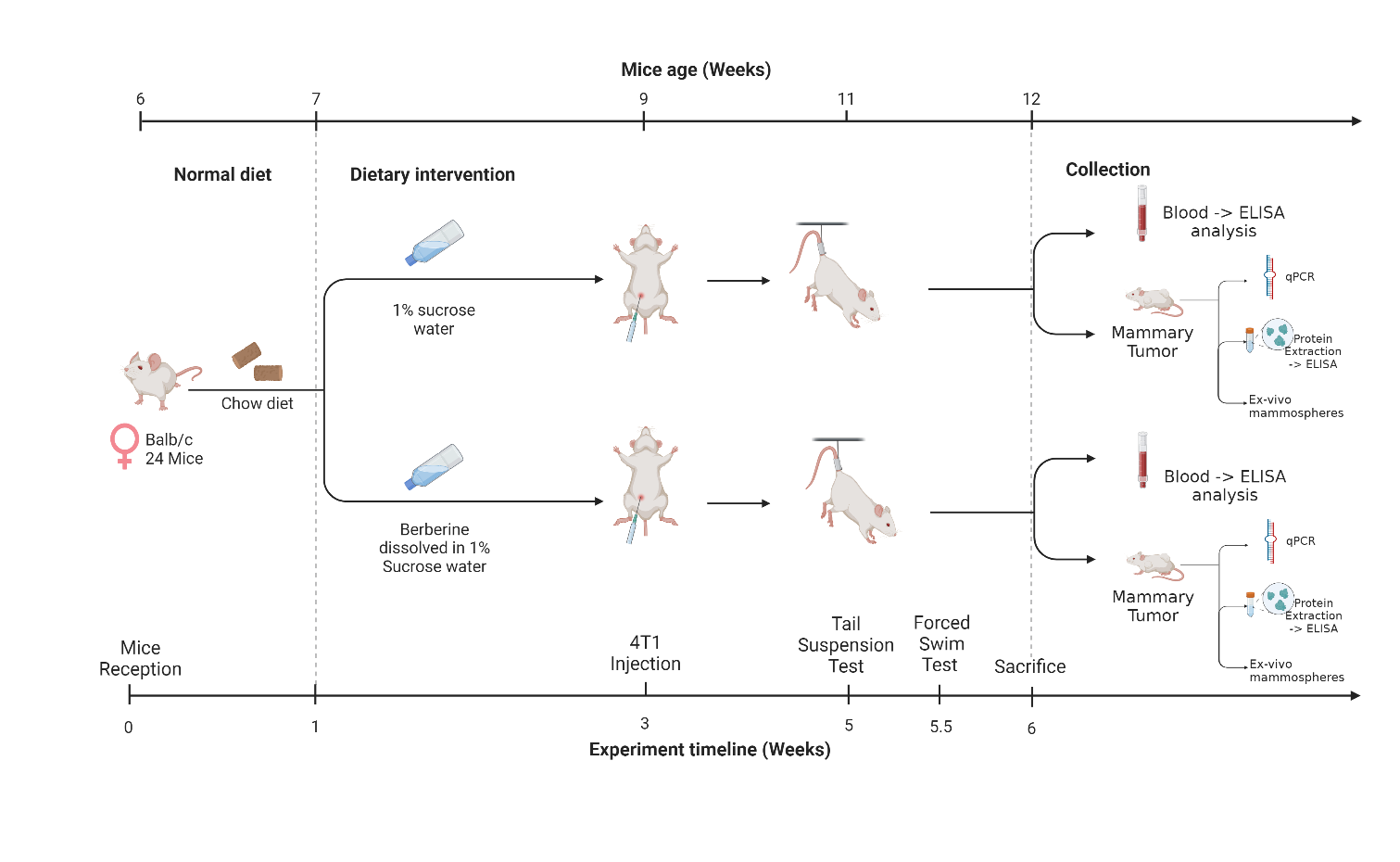


Supplementary Figure 2


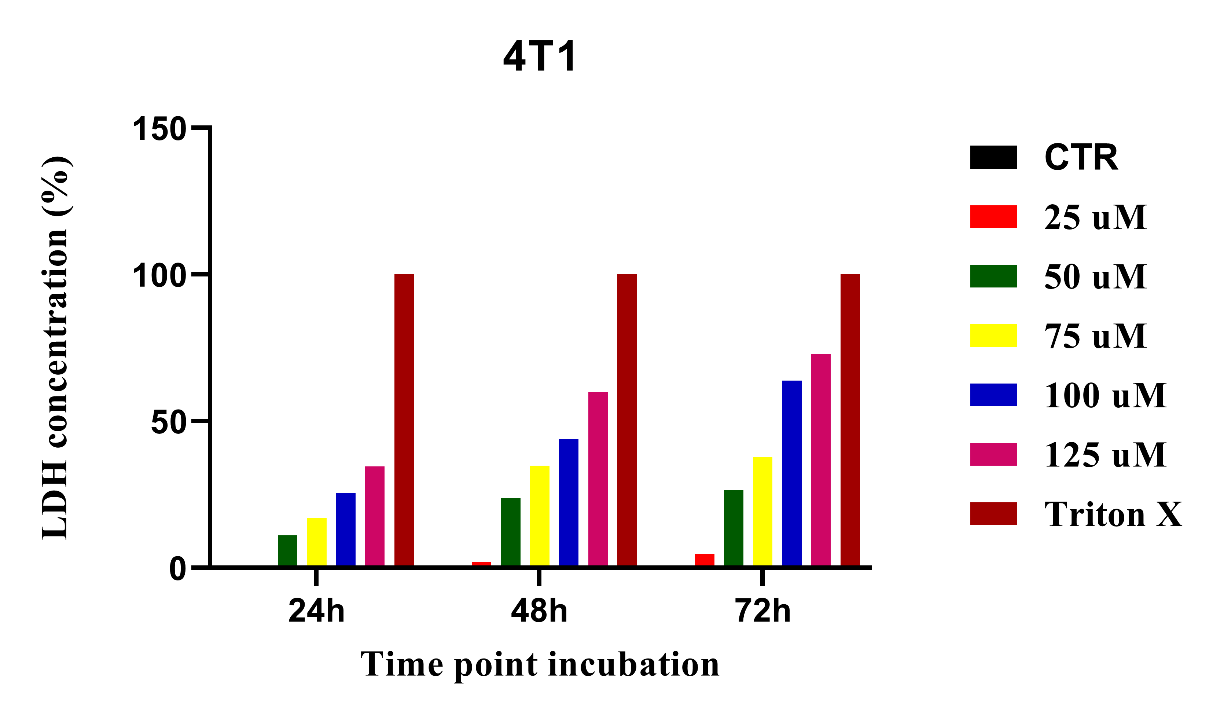


**Supplementary Figure Legend:**

**Supplementary Figure 1:** Experimental timeline for in vivo experiments using berberine in Balb-c mice.

**Supplementary Figure 2:** The Percentage of LDH Concentration at Different Time Points for 4T1 Cell Lines Treated with Various Concentrations of berberine
